# The Blood–Brain Barrier—A Key Player in Multiple Sclerosis Disease Mechanisms

**DOI:** 10.3390/biom12040538

**Published:** 2022-04-02

**Authors:** Thomas Gabriel Schreiner, Constantin Romanescu, Bogdan Ovidiu Popescu

**Affiliations:** 1 Faculty of Medicine, “Carol Davila” University of Medicine and Pharmacy, 050474 Bucharest, Romania; bogdan.popescu@umfcd.ro; 2 Faculty of Medicine, “Gr. T. Popa” University of Medicine and Pharmacy, 700115 Iași, Romania; 3 Department of Electrical Measurements and Materials, Faculty of Electrical Engineering and Information Technology, Gheorghe Asachi Technical University of Iasi, 21-23 Professor Dimitrie Mangeron Blvd., 700050 Iasi, Romania; 4 Clinical Section IV, “St. Parascheva” Infectious Disease Hospital, 700116 Iași, Romania; 5 Neurology Department, Colentina Clinical Hospital, 020125 Bucharest, Romania; 6 Laboratory of Cell Biology, Neurosciences and Experimental Myology, “Victor Babes” National Institute of Pathology, 050096 Bucharest, Romania

**Keywords:** blood–brain barrier, multiple sclerosis, neuroinflammation, neurodegeneration, microglia, immune cells

## Abstract

Over the past decade, multiple sclerosis (MS), a chronic neuroinflammatory disease with severe personal and social consequences, has undergone a steady increase in incidence and prevalence rates worldwide. Despite ongoing research and the development of several novel therapies, MS pathology remains incompletely understood, and the prospect for a curative treatment continues to be unpromising in the near future. A sustained research effort, however, should contribute to a deeper understanding of underlying disease mechanisms, which will undoubtedly yield improved results in drug development. In recent years, the blood–brain barrier (BBB) has increasingly become the focus of many studies as it appears to be involved in both MS disease onset and progression. More specifically, neurovascular unit damage is believed to be involved in the critical process of CNS immune cell penetration, which subsequently favors the development of a CNS-specific immune response, leading to the classical pathological and clinical hallmarks of MS. The aim of the current narrative review is to merge the relevant evidence on the role of the BBB in MS pathology in a comprehensive and succinct manner. Firstly, the physiological structure and functions of the BBB as a component of the more complex neurovascular unit are presented. Subsequently, the authors review the specific alteration of the BBB encountered in different stages of MS, focusing on both the modifications of BBB cells in neuroinflammation and the CNS penetration of immune cells. Finally, the currently accepted theories on neurodegeneration in MS are summarized.

## 1. The Blood–Brain Barrier—Structure and Physiological Functions

**Figure 1:**
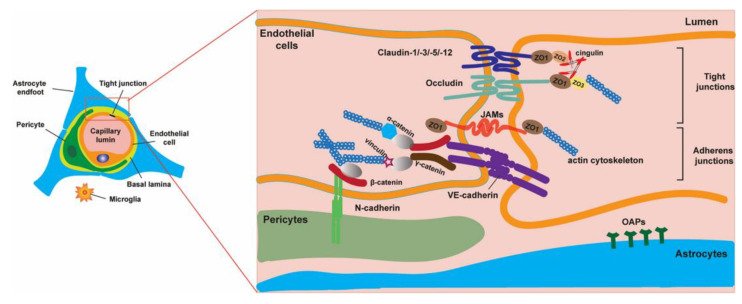
The cellular and most significant molecular components of the blood–brain barrier as part of the neurovascular unit (Reprinted with permission from Kadry, H., Noorani, B. and Cucullo, L. A blood–brain barrier overview on structure, function, impairment, and biomarkers of integrity. *Fluids Barriers CNS* **17**, 69 (2020). https://doi.org/10.1186/s12987-020-00230-3) [4].

Together with the blood-cerebrospinal fluid (CSF) barrier and the arachnoid barrier, the blood–brain barrier (BBB) plays an essential role in maintaining cerebral homeostasis by regulating, in a highly selective manner, the bidirectional exchanges between the circulatory system and the central nervous system (CNS) [1]. The BBB is a complex structure (Figure 1) comprising endothelial cells of the CNS microvessels and of additional structures that are fundamental for its proper functioning [2]. Furthermore, a myriad of circulatory molecules, including inflammatory factors and hormones, are responsible for ensuring the BBB’s physiological selective permeability [3].

### 1.1. Brain Microvascular Endothelial Cells

Several cellular components ensure the structural stability of the BBB, with endothelial cells (ECs) representing the central structural element of the barrier. This type of endothelial tissue is unique, consisting of flattened cells, closely linked together, lining the inside of the blood vessels. ECs are anchored to the basement membrane by cell adhesion molecules (CAMs), belonging to the classes of selectins, the immunoglobulin superfamily, and the integrin family. At the cerebral level, ECs have distinct characteristics, being more specialized compared to ECs located in other organs [5]. Brain microvascular ECs (BMECs) have an increased mitochondrial content [6], a lack of fenestrations [7], minimal pinocytotic activity [8], and the presence of an elaborate network of tight junctions (TJ) and adherens junctions (AJ) between cells [9].

TJs prevent paracellular diffusion and act as a physical barrier to regulate the movement of ions and other molecules between the peripheral blood flow and brain parenchyma. This complex consists of a set of several proteins, the most relevant being the transmembrane proteins (claudin, occludin, and junctional adhesion protein), which, although function independently of each other, work in unison to the effect of joining the plasma membranes of adjacent ECs to seal the paracellular space between them [10]. At the intracellular level, scaffolding proteins zona occludin-1 (ZO-1), ZO-2, and ZO-3 establish a link between the abovementioned transmembrane proteins and the actin cytoskeleton [11,12]. In addition, proteins are also associated with zonula adherens that form the adherens junctions formed by proteins such as the platelet-endothelial cell adhesion molecule (PECAM) and vascular endothelial-cadherin, also called cadherin-5. Forming a zipper-like structure, it regulates intercellular spaces and maintains BBB integrity [13].

Among the many claudin family members, claudin 5, 11, and 12 were reported to be found in the CNS [14]. Occludin, the first transmembrane protein identified at the TJ level, has a high molecular mass and complex structure, its intracellular domains interacting with zonula occludens (ZO) proteins in order to stabilize the TJ [15]. The presence of occludin in other areas of the body (gastric epithelium, testis, bone) and its changes in various pathologies suggest that occludin has other undiscovered roles, along with the simple structural role in the TJ component [16]. Finally, junctional adhesion molecule (JAM) proteins also enter the molecular structure of these structures, interacting with important intracellular elements, presumably zonula occludens (ZO), the structure that in turn interacts with actin, to maintain cytoskeletal integrity, and thus to ensure BBB impermeability [17].

While the TJs at the BBB greatly restrict the paracellular diffusion of many substances from the blood, small gaseous molecules such as O_2_ and CO_2_ can diffuse freely through lipid membranes [18]. The presence of specific transport systems on the luminal and abluminal membranes of the capillaries regulates the transcellular traffic of essential molecules between the bloodstream and brain; these include transporters and/or receptors for nutrients [19] and proteins such as insulin, leptin, and transferrin [20]. Restrictive transport across the BBB also results from reduced transcellular transport [21]. Transcytosis is the main process that ensures the transit of both smaller and larger molecules [21], being altered in physiological conditions such as aging [22] and during pathological states [23].

BBB endothelial cells also serve the role of transporting molecules against their concentration gradient. This is achieved via members of the well-known ATP-binding cassette (ABC) transporter superfamily in an energy-dependent process. Important subfamilies of transporters aiding the process of cellular efflux include the permeability glycoprotein (P-gp) and the multidrug resistance protein (MRP) transporters which are found on the luminal side of ECs, albeit there is recent research reporting the presence of transporters on the subendothelial side as well [24]. On the luminal side, MRP1 and MRP2 appear to carry out the translocation of organic non-polar negatively charged ions and HIV protease inhibitors, whereas MRP4 and MRP5 transport cyclic nucleotides alongside organic anions and weak acids [25,26]. Moreover, as part of the ABC transporter superfamily, breast cancer-related proteins (BCRPs) pump out both organic cations, and negatively charged drug molecules, as well as weak organic bases with non-polar regions [27].

Although BMECs with their morphological features represent the physical structure of the barrier, it should be noted that maintenance of the BBB is dependent upon the normal functioning of astrocytes (contacting the brain capillaries through numerous endfeet), pericytes (embedded within the basement membrane that they share with ECs), perivascular microglia, and the basal laminae, which are found in close proximity to the capillary and postcapillary venules of the CNS [28,29].

Therefore, for some years now, the term neurovascular unit (NVU) has been preferred, designating, on the one hand, the fact that several cells enter the BBB component, each having a special role in maintaining the quality of the barrier microenvironment, and, on the other hand, that any pathological change will involve alterations in the function and structure of these components [30,31].

### 1.2. Smooth Muscle Cells and Brain Pericytes

Smooth muscle cells (SMCs) make up the middle tunic of the blood vessel and, together with the brain pericytes, cover the vascular endothelium. SMCs are contractile cells present in arterioles and venules, whereas brain pericytes are perivascular cells adjacent to brain capillaries belonging to the lineage of SMCs [32]. Brain pericytes are an essential element in the induction and maintenance of the BBB properties [33], increasing the stability of the EC monolayer, and promoting the formation of TJ from the prenatal phase forwards [34]. Pericytes also appear to participate in the metabolic properties of the BBB by inducing the expression of enzymes in ECs [35] or by enhancing the expression of some efflux pumps (MRP6) [36]. Besides modulating vascular contractility, pericytes also synthesize type IV collagen, glycosaminoglycans, and laminin, important components of the basement membrane [37].

Pericytes contribute to the neuroimmune response and are potent modulators of BBB function due to their proximity to ECs. The close brain pericyte–EC association translates into maintaining viable TJ and a low level of transcytosis and CAM expression, thus including the very strict control of leukocyte transshipment at BBB level in physiological conditions [38]. Once the intercellular crosstalk relationships are established, both the pericytes and the EC will produce transforming growth factor beta (TGF-β) which, in turn, triggers the production of adhesion molecules such as laminin, while in the BMECs, it induces cadherin-2 (also known as N-cadherin) that promotes the adherence of pericytes [39]. Moreover, pericytes secrete cytokines and chemokines in cell culture and upregulate cytokines in response to lipopolysaccharide (LPS) [40,41]. They also present antigens in response to interferon (IFN)-γ, which may contribute to T-cell activation [42].

Pericytes serve to protect the endothelium of blood vessels and also ensure high BBB selectivity by sustaining vascular proliferation and development, as well as membrane hyperpolarization. This is thanks to the “intercellular crosstalk” with the other elements of the NVU [43]. New studies [44] question the contractile capacity of brain pericytes to the detriment of SMCs in the arteries and brain capillaries. To ensure neurovascular coupling, it is necessary to regulate blood flow according to the needs of the neuron, as there are variations according to location. The main cells that increase or decrease the vascular lumen remain the SMCs, which regulate blood flow in both healthy and pathological cases. Arteriolar SMCs are arranged on the circumference of the vessel, forming similar structures to bands [34]. Moreover, arteriolar SMCs have been found to express α-SMA in both human and mouse brains, explaining their contractile properties.

### 1.3. Astrocytes

The abundance of astrocytes in the brain parenchyma, as well as their location, suggest that glial cells have an important role in the NVU. Within the NVU, astrocytes are located in a position (between neurons, pericytes, and capillary ECs) that allows them to both receive neuronal input and communicate intercellularly with the endothelium. Thus, one of the roles is to provide support for the maintenance and repair of surrounding structures, as a great number of chemical mediators which promote the BBB phenotype, such as TGF-β, glial-derived neurotrophic factor (GDNF), basic fibroblast growth factor (bFGF), and angiopoetin-1 (Ang1), are also secreted by astrocytes [45]. Perivascular astrocyte endfeet wrap around endothelial cells and help regulate brain water transport primarily through the expression of aquaporin-4 [46]. Through this structure, the fluid flow at the CNS level is regulated, as is the excretion of toxic substances [47].

Additionally, astrocytes are also able to regulate the expression and localization of various transport proteins and enzyme systems on the endothelium [18]. Experiments using in vitro BBB models (endothelial cells cultured on filter in the presence of astrocytes) or conditioned medium have been shown to induce barrier properties by increasing trans-endothelial electrical resistance [48], or the expression of efflux pumps such as P-gp and some MRPs [36]. On the other hand, some studies suggest endothelial cells secrete factors involved in astrocyte growth and differentiation [49], further underscoring the importance of signaling between astrocytes and endothelial cells. ECs are thought to produce short peptides called endothelin that bind to specific receptors, promoting the production of key factors such as brain-derived neurotrophic factor (BDNF), GDNF, nerve growth factor, and neurotrophin-3 [50].

### 1.4. Oligodendrocytes

Although not directly involved in the structure of the BBB, the oligodendrocyte and the oligodendrocyte progenitor cell (OPC) seem to play an important role in maintaining BBB integrity. The main function of oligodendrocytes at the CNS level is to ensure correct myelin insulation of the axons. However, several recent studies have highlighted the additional capability of these cells to modulate BBB tightness via different mechanisms. For example, Seo et al. [51] revealed that OPCs are able to upregulate TJ proteins via TGF-β signaling, while Wang et al. [52] suggested Wnt/β-catenin to be the main regulatory pathway for claudin-5 expression in ECs. Extensive research related to the osmotic demyelinating syndrome revealed interesting data regarding the crosstalk between oligodendrocytes and the other cellular components of the BBB. Thus, the intercellular connections of oligodendrocytes with astrocytes situated in their proximity are essential for myelination activity [53], with studies suggesting that BBB leakage leads to damage in astrocytes as the first step [54], with oligodendrocyte alteration being a subsequent phenomenon. Multiple molecular mechanisms including the imbalance between protein synthesis and degradation [55], the impact of aquaporin-1 and aquaporin-4 [56], and finally the role of ionic equilibrium [57] have been proposed; however, the complete role of oligodendrocyte in the maintenance of BBB physiological function remains to be fully explained in the future.

### 1.5. Microglia

Microglia are considered to be the equivalent of immune cells in the CNS, being both an integral part of NVU structure, and serving the function of immune surveillance. From an embryological point of view, microglia are found in future CNS before EC migration, participating in the modulation of cerebrovascular development [58]. In the case of inflammation, microglia is the key player that will subsequently generate the cellular and humoral inflammatory response, with all the cascade of related reactions.

The link between microglial cells and BMECs and their effects in regulating BBB properties are incompletely explained. Microglia, the cells derived from hematopoietic precursors that migrate into the CNS parenchyma, have a primary immune and defense role in the BBB [59]. In this context, research thus far has focused on the role of microglia in CNS inflammation. Although representing only 10% of the cell population in the CNS, microglia are found in both white and gray matter, playing a central role in neuroinflammation. When these cells are activated, they lose their long processes, while expressing inflammatory cytokines (IL-1, tumor necrosis factor alpha (TNF)), chemokines, prostaglandins, and reactive oxygen species [60]. In addition, the transformation of microglial cells can theoretically follow two pathways, turning into the M1 (pro-inflammatory) or M2 phenotype (with anti-inflammatory valences by releasing vascular endothelial growth factor (VEGF) and chemokines that help repair tissues); however, newer research suggests a very fine border between the two states [61].

### 1.6. Neurons

In addition to the above-mentioned glial cells, neurons also exert an important direct influence at the BBB level. It is believed that neurons are at a maximum distance of 25 μm from capillaries, being in the vicinity of endothelial cells [62]. Thus, they are dependent on changes in the microenvironment, especially in terms of ionic composition [63]. Neurons regulate blood flow and BBB permeability by promoting tight junctions and downregulating efflux transport [64].

### 1.7. Extracellular Matrix

Within the NVU there are also relevant non-cellular elements, such as the extracellular matrix (ECM) or basement membrane of capillary cells, whose physiological role is not totally elucidated, but which undergoes changes in case of inflammation, for example, favoring increased BBB permeability and penetration of cells, and potentially toxic substances at the CNS level [65].

The cerebral capillary basement membrane remains the object of study of numerous papers in this area of research, the distinct feature being the increased presence of proteins, at least 30 different types, of which the most abundant are contractin-1, perlecan, agrin, and laminin [66]. Two structurally distinct basement membrane (BM) structures, the vascular BM and the parenchymal BM, secreted by pericytes and astrocytes, respectively, serve the function of a barrier against immune cell entry to the CNS [67]. Laminin α4 and α5 are major components of the vascular BM, whereas laminin α1 and α2 contribute to the structure of the parenchymal BM [68]. These are thought to play a role in controlling/regulating BBB function, including CD4 + lymphocytes acting differently on each membrane [69].

Another barrier to blood cells’ entry into brain parenchyma during inflammation and hemorrhage is the ECM [70]. ECM breakdown by metalloproteinases (MMPs) may contribute to BBB disruption, with consequent leukocyte trafficking, a mark of CNS inflammation [71]. Hyaluronan and its fragments bind Toll-like receptors to favor the neuro-immune environment [72].

### 1.8. The Physiological Roles of the BBB

Having a surface area between 12 and 18 m^2^ in adults, the BBB is essential for the bidirectional blood–brain exchange of substances [73].

As the name suggests, the BBB is a real barrier to many substances found in the blood, perhaps beneficial to other organs, but with potentially detrimental effects for sensitive brain tissue [74]. Among the substances that do not physiologically penetrate the BBB are macromolecules; their increased size, along with the polarity of the molecule, are some of the restrictive characteristics of the barrier passage [75]. In connection with this limitation of the entry of numerous substances at the CNS level, we also note the protective role of the BBB. Against both endogenous toxins, resulting from metabolism, and exogenous toxins that penetrate the human body, defense mechanisms enter the circulatory system and have potentially neurotoxic effects [76].

In neurophysiology, ion homeostasis is maintained by the BBB [77]. Specialized protein structures, alongside the Na-K pump, maintain the intra-/extracellular concentration of various electrolytes such as Na, K, Ca, Mg, and Cl—crucial for neuronal metabolism, electrophysiology, and overall adequate cell function [78].

Regarding metabolites and other substances essential for maintaining neuronal functions, these will cross the BBB via passive mechanisms such as diffusion down a concentration gradient, or via the active transport of liposoluble molecules [79].

Another function of the BBB is to adjust and maintain optimal levels of neurotransmitters within the CNS. If out of balance, some neurotransmitters can become neurotoxic. For example, glutamate in high concentrations can irreversibly destroy brain tissue by inducing neuronal death in the penumbra [80].

Finally, another important function of the BBB is that of eliminating toxic and residual end-products resulting from neuronal metabolism. Toxic end products cross the BBB to be released into the bloodstream, and subsequently for delivery to the kidneys or the digestive tract where excretion occurs [81].

Thus, the structurally intact BBB serves crucial roles in CNS physiology. Consequently, any local or systemic disorder that leads to inflammation directly or indirectly alters the structure of the BBB and potentially results in CNS damage (Table 1).

## 2. The Blood–Brain Barrier in Multiple Sclerosis—From Early Neuroinflammation to Neurodegeneration

BBB dysfunction is an early feature of several CNS pathologies such as autoimmune inflammatory diseases, CNS infections, and neurodegenerative diseases [82]. Multiple sclerosis (MS), a chronic neuroinflammatory CNS disorder, is currently affecting more and more people, with increasing incidence and prevalence observed worldwide [83]. Although many therapeutic options have emerged during the last two decades [84], no curative treatment is currently available. In this context, it is essential for researchers to better understand MS pathogenesis and the role of BBB breakdown in disease onset and evolution.

Two main pathological hallmarks are related to MS: early-stage chronic neuroinflammation, and neurodegeneration in the later stages and in particular subtypes of the disease, such as SPMS. Inflammation at the CNS level, also known as neuroinflammation, includes both positive and negative elements for the brain and spinal cord. Among the beneficial aspects, we mention immune surveillance, neuroinflammatory signaling by cytokines such as interleukin-1 (IL-1) and interleukin-4 (IL-4) that help memory and learning, remodeling by inflammation through the M2 microglia and, finally, eustress and “good inflammation” [85]. Another disorder, osmotic demyelinating syndrome, may offer relevant data related to the abovementioned concepts, as ionic rebalancing is the treatment approach for BBB disruption encountered within this syndrome, raising questions as to whether neuroinflammation has a real beneficial impact on pathological CNS conditions.

**Figure 2:**
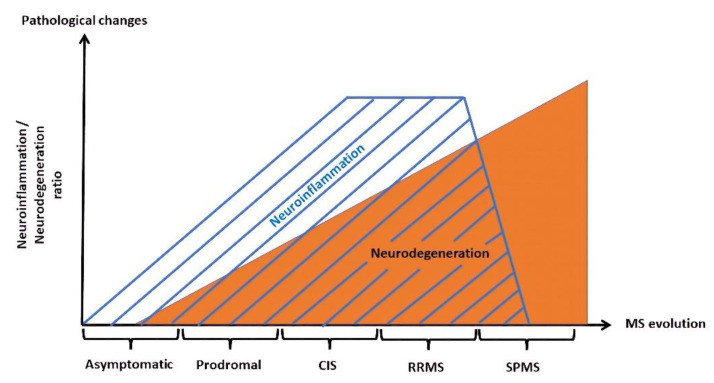
Neuroinflammation and neurodegeneration in multiple sclerosis.

Neuroinflammation is more frequently associated with its negative aspects, such as those associated with early-stage neurological diseases, including early-stage MS (Figure 2).

### 2.1. Consequences of Neuroinflammation at the BBB Level

The BBB is the main structure altered in neuroinflammation, losing its permeability as a result of astrocyte activation, excessive production of cytokines and chemokines, and immune cell infiltration [86]. All cellular and non-cellular components of the BBB are damaged in inflammatory conditions [87] (Table 2).

#### 2.1.1. Alteration of ECs during Inflammation

The alterations of ECs in inflammation are not limited to intracellular changes, and thus the endothelium must be seen as a whole and considered as a single component. Inflammatory mediators exert their effects on several levels, intervening with and affecting multiple processes. Firstly, they induce paracellular BBB leakage by modulating TJ and AJ proteins. While in physiological conditions ECs are impermeable because of the existence of TJ and AJ, in the setting of inflammation, these connections will be altered, resulting in unwanted BBB permeability [88]. Thus, by increasing the production of VEFG-A, certain inflammatory mediators (IL—1β, IL—6, IL—17, IFN—γ, TNF—α, and chemokine (C-C motif) ligand 2 (CCL2)) will reduce the expression of TJ proteins, such as occludin, claudin—5, ZO—1, and JAM—A, or change the location of these junctional molecules and therefore disrupt the integrity of the BBB [89]. Among the structural changes recorded in the affected TJ proteins, we mention hyperphosphorylation, which will cause alteration of the secondary and tertiary structure of the molecule, with the detachment of the protein chains [90].

Inflammatory mediators can also modulate transcytotic vesicular pathways, causing increased BBB permeability. The complex inflammatory process also alters other characteristics of BMECs, such as changing the concentration and function of different membrane receptors (mainly Toll-like receptors). In so doing, transcellular transporting mechanisms ensure the greater extravasation of immune cells (neutrophils, macrophages, lymphocytes). Regarding receptors and signaling pathways, Toll-like receptors (TLR) are one of the most intensively studied families, with research demonstrating their existence in the physiological conditions of TLR2, TLR3, TLR4, and TLR6 on both rat and human cerebral endothelial cells [91]. During inflammation, significant upregulation is observed, mediated by oxidative stress and TNF-α. More precisely, in the experiments conducted by Nagyoszi et al. [91], reactive oxygen species (ROS) equivalents induced a more than 5-fold increase in the expression of TLR2 and TLR3, an almost 4-fold increase in TLR4, and up to a 10-fold increase in TLR6. TLR2 and TLR3 were also strongly upregulated to TNF-α stimulation by about 10 and 8 times, respectively. The results are in line with other findings in ECs with different localization. For example, inflammatory stimuli also induce the expression of TLR2 receptors in human airway epithelial cells [114], TNF-α being the key player, activating TLR2, TLR4, and TLR6 by their specific ligands, which subsequently can increase the production of TNF, leading to a vicious circle [115]. There is increased vacuolar transendothelial transport as a result of increased pinocytosis [116]. Changes in the function of BMECs lead to the increased production of adhesion molecules and the production of VEGF, which subsequently increases permeability [117]. VEGF’s main function is to increase angiogenesis, increasing blood vessel permeability as part of its angiogenic properties. The increased expression of vascular adhesion molecules on the surface of BMECs means greater leukocyte infiltration into the brain, which maintains inflammation. The infiltration process requires interaction with adhesion proteins in BMECs (i.e., selectins) and anchoring and docking proteins (i.e., Intercellular Adhesion Molecule 1 (ICAM-1)) [92]. Furthermore, in severe cases, inflammation can irreversibly damage BMECs, leading to cell death. Apoptosis is believed to play a role, being reported in animal models of ischemia and in the deprivation of oxygen and glucose [118].

An increase in BBB permeability in the setting of inflammation is also believed to occur due to the modulation of several important transporters (such as the members of the solute carrier superfamily) by inflammatory mediators. In vitro studies have demonstrated a predominantly stimulatory effect of pro-inflammatory cytokines on glucose utilization. In this context, enhanced IL-1β expression and nitrous oxide (NO) production found in hypoxia stimulated the expression of the GLUT1 mRNA [93]. Other glucose transporters are also modulated, as another report has shown that LPS endotoxin enhanced GLUT3 [119]. Moreover, amino-acid transport at the BBB level suffers alteration in inflammatory states, as shown in a recent paper assessing the increase in tryptophan uptake by the transport mechanisms of tryptophan in brain capillary endothelial as an effect of neuroinflammation (LPS and TNF-α as main triggers) [120]. Another in vivo study [94] demonstrated the downregulation of L-type amino acid transporter 1 (LAT1) in inflammatory states, with the thyroid hormone passage being modulated similarly to other important biomolecules (adenosine, insulin) in neuroinflammation. Efflux transporters are also modulated, with P-gp expression and activity suffering changes in cases of neuroinflammation. In vitro, BBB P-gp activity was found to be downregulated after short-term exposure to inflammatory mediators, whereas its activity was upregulated following more prolonged exposure. Results from in vivo studies on human and animal subjects have suggested a link between CNS inflammation, peripheral inflammation, related clinical neuroinflammatory disorders, and alterations in the expression and activity of P-gp at the BBB level [95].

Lastly, subsequent to the degradation of the glycocalyx which occurs during inflammatory states, an increased passage of solutes across the endothelial barrier together with increased leukocyte adhesion to the endothelium were observed [121]. During prolonged inflammatory states, the glycocalyx suffers degradations caused by inflammatory factors, such as cytokines, metalloproteinases, heparinase, and hyaluronidase. Many pre-clinical and clinical studies have demonstrated the association between TNF-α, IL-1β, and IL-6 and an increasing trend in glycocalyx destruction markers (heparin sulfate, hyaluronan, syndecan-1). Nieuwdorp et al. [96] administered endotoxin followed by soluble TNF-α receptor etanercept in healthy male volunteers. By measuring endothelial glycocalyx thickness and other related parameters such as hyaluronan, the authors were able to quantify the amplitude of perturbation and destruction caused by TNF-α at the glycocalyx level. Results were in line with studies conducted on mice, where TNF-α alone, via heparanase activation, was sufficient to induce glycocalyx degradation in septic mice [122]. Elevated levels of IL-6, along with other inflammatory cytokines have also been reported in association with increased glycocalyx destruction, with an up to 2.5-fold increase according to a recently conducted observational trial on patients undergoing cardiac surgery with a cardiopulmonary bypass [123].

Other inflammatory markers that contribute to glycocalyx destruction include the proteolytic enzymes of the MMPs group. MMPs, in the setting of oxidative stress, exert their effects on glycocalyx to increase the expression and proteolytic activity of MMP-2 and MMP-9. This subsequently increases the shedding of syndecan-1 and limits extracellular superoxide dismutase (SOD3) activity [124]. Among glycocalyx destruction markers, glycosaminoglycans (GAGs) represented by heparan sulfate and hyaluronic acid are directly associated with inflammation and destruction intensity, respectively. Thus, because increased plasma levels are encountered in septic shock patients, with plasma levels being correlated to survival prognosis and inflammation severity, heparan sulfate and hyaluronic acid have been reported as having potential biomarker roles in the near future [125].

#### 2.1.2. Pericytes Modification in Inflammatory Conditions

The pericyte to EC ratio of the cerebral capillary is increased compared to similar structures outside the CNS, where permeability is less regulated.

The pericyte also maintains and protects the integrity of the BBB through the secretion of various substances. Some of these include Ang-1, TGF-β1, and other factors involved in the maintenance of the basement membrane [126]. In the setting of inflammation, IL-1β and TNF-α will initiate the inflammatory cascade with downstream synthesis and the release of other proinflammatory cytokines that, in turn, exert their effects on the pericyte, as has been observed in HIV pathology studies [127] and in porcine brain pericytes [97].

In MS animal models, the administration of TNF-α resulted in the modification of pericyte α1/α2 integrin levels, with a subsequent downregulation of α-SMA and slowdown of cell differentiation [98].

Inflammation of the CNS leads to pericyte detachment from ECs with subsequent transformation into phagocytic or fibroblast cells. This pattern is associated with an increase in the number of EC vesicles, TJ disruption, and immune cell recruitment [128]. Furthermore, neuroinflammation is associated with fibrosis, the accumulation of pericyte-derived fibrin and scarring, and neurotoxic consequences that lead to cellular death [43]. Another indicator of pericyte differentiation into fibroblasts is fibroblast activity as measured by fibrin deposits.

The precise mechanisms whereby pericytes moderate inflammation in the CNS have yet to be understood, however. It remains unclear how pericytes interact with leukocytes to aid in their migration across the endothelial barrier. Pericyte detachment from the basement membrane and subsequent acquisition of phenotypical characteristics reminiscent of infiltrating macrophages may play a role [99]. In summary, pericytes appear to contribute to the neuroinflammatory response via: (1) favoring a leaky BBB by secretion of endothelial-disrupting factors or by physical detachment; (2) favoring transport of immune cells and pathogens into the brain; (3) by creating a pro-inflammatory environment locally and also through active recruitment of immune system cells to the site of inflammation.

#### 2.1.3. Astrocyte Modification in Inflammatory Conditions

Through close contact with EC, under physiological conditions, the astrocyte maintains the functional integrity of the BBB and attenuates its damage. This function is possible by producing special substances (astrocyte-derived factors), whose release is inhibited or exacerbated in response to inflammation, with subsequent consequences at the level of BBB permeability [129].

Glutamate, produced mainly by neurons and to a lesser extent by astrocytes, despite being essential in the normal functioning of the CNS, when in excess (cerebral ischemia), will lead to excessive permeability of the BBB following NMDA receptor activation [100].

Protective factors produced by astrocytes have the role of counteracting the destructive effects of the astrocyte factors listed above. Thus, Ang-1 (even exogenously administered in excess) has a protective effect on BBB, causing the upregulation of ZO-1 and occludin in order to repair TJs after permanent ischemic damage in rat models [101]. Ang-1 also suppressed the VEGF-induced expression of ICAM-1 and vascular cell adhesion molecule 1 (VCAM-1), and reduced VEGF-induced leukocyte adhesion to human umbilical vein endothelial cells (HUVECs) [130]. Another protective factor is sonic hedgehog (Shh), which has an anti-apoptotic role in endothelial cells. There is also an effect at the TJ level, where Shh increases the expression of claudin-5, occludin, and ZO-1, promoting the restoration of BBB impermeability [102]. Glial-derived neurotrophic factor (GDNF) and retinoic acid act similarly to TJ proteins, stimulating the production of occludin and ZO-1. Insulin-like growth factor-1 (IGF-1), produced by astrocytes, but also found in other cells of the BBB, helps primarily to maintain the neuronal viability of endothelial cells, maintaining the intact structure of the BBB [131].

Through the released factors, the astrocyte also regulates the level of expression of the endothelial ICAM-1 and VCAM-1 molecules, which interact with integrin α4β1 (VLA-4) and lymphocyte function-associated antigen 1 (LFA-1) in leukocytes. In the context of inflammation, an increased expression of ICAM-1 and VCAM-1 will favor the penetration of leukocytes into the CNS.

#### 2.1.4. Oligodendrocytes and OPCs in Neuroinflammation

Oligodendrocytes and their precursors (OPCs), known to be key factors in the early stages of MS (regional CNS demyelination), have recently gained attention in the context of chronic neuroinflammatory state related to BBB leakage. The intricate multidirectional cellular crosstalk among OPCs, astrocytes, and pericytes is of increasing importance, with cytokines and other inflammatory mediators being the main links within this microenvironment. During neuroinflammation, there is an increased expression of inflammatory genes together with increased phagocytosis in oligodendrocytes, mainly as a result of IFN-γ, IL-6, and IL-1 produced by other BBB cells such as astrocytes or microglia [53]. Another relevant aspect is the increased apoptosis of mature oligodendrocytes, while OPC maturation is heavily suppressed. Finally, newer hypotheses pointing towards oligodendrocyte dysfunction as the missing link between neuroinflammation and neurodegeneration (particularly in progressive MS), are incompletely understood and necessitate further scrutiny [103].

#### 2.1.5. Microglia Activation in Inflammation

In the early phase of acute inflammation, the microglia are activated (within minutes) of the initiation of the CNS charge, the response being a long one, taking up to several weeks. This activation of the microglia means important changes both in terms of cellular phenotype and in terms of secreted substances. Known as the M1 proinflammatory phenotype, the cell will express Iba-1 on its surface and as exogenous products, reminiscent of the heterogeneous group of proinflammatory cytokines (IL-1, IL-6, TNF-α), chemokines (CCL2, C-X3-C motif chemokine ligand 1 (CX3CL1), and macrophage inflammatory protein 1 (MIP-1)), MMPs, and oxygen free radicals [104]. After overcoming the acute phase, the microglia phenotype changes into the anti-inflammatory, phagocytic M2-type, which will lead to angiogenesis and neuroprotection [132].

The BBB-microglia link becomes apparent when considering modifications in the structure and permeability of the BBB resulting from inflammation where microglia play a central role. Microglial activation occurs following neuronal damage, under the action of various damage-associated molecular patterns (DAMPS), a heterogeneous group of protein compounds such as S100 heat-shock proteins [133] or high-mobility group box 1 (HMGB-1) [134], nucleic acids, and ATP acids, and even cytokines such as neuron-derived fractalkines (CX3CL1) [105]. Even elements resulting from the destruction of ECs and the basement membrane of ECs could be DAMPS, leading to activation of microglia [135]. In addition, numerous studies have shown a direct link between microglial dysfunction, neuroinflammation, and the subsequent occurrence of neurodegenerative pathologies such as Alzheimer’s disease (AD) or Parkinson’s disease (PD) [136]. Thus, microglial activity is related to multiple AD modifications, from lipid transport to alterations in transmembrane proteins, cytoskeletal dynamics, and transcription factors. For example, *ApoE* microglial expression is encountered, explaining the deposition of amyloid beta (Aβ) in altered AD brain. ABCA7, an ATP-binding cassette transporter is presumed to play a role in membrane remodeling which, when altered, is correlated with impaired Aβ phagocytosis in mice [137]. Another example is the IL-1 receptor accessory protein (IL1RAP), a coreceptor with IL1R1 for IL-1 signaling, which enables proinflammatory signal transduction that may exacerbate Tau pathology [138]. Finally, research has also revealed the possible role of the myocyte enhancer factor 2C (MEF2C) transcription factor expressed by the microglia in downregulating the triggering receptor expressed on myeloid cells 2 (TREM2) [139].

The bidirectional relationship between microglia and the rest of the BBB components, both in physiological conditions and in acute or chronic inflammatory pathology, supports the central role of microglia in inflammation. One crosstalk pathway between microglia and the neuron is via fractalkine signaling, with the soluble CX3CL1 compound presumed to keep the microglial phenotype in a neuroprotective state. The disruption of CX3CL1–CX3CR1 signaling leads to microglial response dysregulation and neuronal damage. In AD, this pathway is supposed to be affected differently in the early versus later stages of the disease. In early AD, Aβ accumulation causes a mild decrease in neuron–microglia crosstalk via CX3CL1–CX3CR1 signaling, leading to the enhanced microglial phagocytosis of Aβ. As the disease progresses, the neuron–microglia intercommunication is further exacerbated, causing the dysregulation of microglia and abnormal excitation of the neuron, with subsequent neuron damage and loss [107].

Four theories have been postulated in an attempt to explain the link between BBB destruction and microglial activation: (1) the link factors result from the alteration of BBB cellular elements (EC, astroglia) to microglia [135]; (2) the link factors result from the destruction of pericytes and noncellular components of the BBB to microglia [128]; (3) systemic inflammatory markers pass the altered BBB and activate microglia [138]; and (4) chronic changes in the BBB stimulate the microglia (an effect known as microglia priming).

The effects being bidirectional mean that microglia also produce numerous inflammatory mediators, which influence BBB homeostasis. The most relevant cytokines produced by activated microglia appear to be IL-1 and TNFα. Microglia produce both IL-1α in the acute phase of inflammation (e.g., the first hours after stroke), and IL-1beta, the central element that increases BBB permeability [140]. A similar effect is TNFα produced by microglia, resulting in the downregulation of occludin with a subsequent increase in BBB permeability [141]. On the other hand, M2 microglia produce the same factors which, in this case, have a repairing, proangiogenic effect [106]. Important human M2 microglial markers are CD163 involved in the binding and internalization of the hemoglobin–haptoglobin complex [142], or TREM2, which is thought to be involved in debris clearance [143]. Other markers, such as arginase I implicated in tissue remodeling and wounding healing, or chitinase-like protein 3 (Ym1) likely involved in the degradation of extracellular matrix components [144], were detected in murine microglia, with studies in human microglia still in progress.

#### 2.1.6. Immune Cells, BBB, and Inflammation

BBB structure and function also seem to be altered in the pathological processes which stimulate the migration of different populations of myeloid cells, neutrophils, monocytes, and mast cells [145]. Neutrophils, once in the perivascular space, begin to secrete pro-inflammatory cytokines (IL-1β, IL-6, IL-12, TNF-α, and IFN-γ), with different roles in maintaining inflammation. For example, IL-1β activates local antigen-presenting cells (APCs) which, in turn, reactivate encephalitogenic T cells [111]. T lymphocytes can also be stimulated by reactive oxygen species produced by activated neutrophils [146]. IL-1β also acts on the astrocyte, leading to increased VEGF-A production, resulting in altered TJ, resulting in increased permeability of immune cells and inflammatory mediators to the CNS [147].

Regarding monocytes, their transfer through the BBB is dependent on the homophilic interactions of activated leukocyte cell adhesion molecules (ALCAMs), JAM-A, PECAM-1, and CD99 which, in certain pathologies (e.g., HIV encephalitis), are upregulated [148]. Some monocytes (CD14 +), once migrated through the BBB, will change their phenotype to CD83 + CD209 + under the influence of TGF-β, promoting the differentiation of Th1 and Th17 cells [112]. Mast cells found physiologically in the meninges, choroid plexus, or hypothalamic region, when activated, secrete inflammatory mediators such as histamine, chymase, tryptase, TNF-α, IL-6, and IL-13, directly or indirectly affecting BBB permeability [113]. Histamine acts on H2 receptors at the BMEC level, increasing the permeability of the barrier. TNF-α produced by mast cells promotes neutrophil recruitment and focal ischemia in the CNS [149]. Compounds such as various environmental factors, oxidative stress and many yet to be studied, likely favor the initiation and maintenance of inflammation in the CNS; these will be detailed in another review.

Finally, the role T and B lymphocytes play in promoting neuronal damage in MS has been the focus of several studies, as MS was considered a T-cell mediated disease for a long time [150]. This hypothesis led to the development of one of the most used primary models that induce MS, the experimental autoimmune encephalomyelitis (EAE) model [151]. As members of both MHC class I and II molecules are risk factors for MS, there are also distinct populations of T cells involved in the demyelinating process. One important population consists of the CD4 myelin-reactive T cells which, compared to healthy controls, are more likely to be in an activated state and display a T helper (Th)1 phenotype in MS patients [108]. IFN-γ and TNF-α are the main proinflammatory cytokines secreted by Th1 CD4 T cells, being directly involved in the activation of local glial and antigen-presenting cells (APCs). Another relevant CD4 T cell fraction for MS includes the Th17 phenotype; however, this is found in a much lower proportion than Th1 cells in CSF and peripheral blood [152]. Th17 CD4 T cells produce other important inflammatory factors, including IL-17, IL-21, and IL-22, that subsequently can either promote the expression of other proinflammatory cytokines such as IL-6, which sustain the inflammatory cascade or stimulate the production of granzyme B that directly kills neurons through the glutamate receptor (GluR3) [153].

Another type of T cell that also plays a relevant role in MS pathogenesis is the CD8 T cell fraction. On one hand, CD8 lymphocytes act within MS patients via their well-known cytotoxic function by introducing granzymes into the cytosol of target cells [109]. Several studies in pathology have demonstrated the abundance of CD8 T cells within parenchymal lesions, with reports suggesting a positive association between their rate of detection in plaques, CSF, and peripheral blood and the intensity and destruction of MS lesions [154]. On the other hand, some studies suggest that particular subsets of CD8 cells are able to secrete cytokines, mainly IFN-γ and IL-17, which subsequently directly kill oligodendrocytes [155].

During the last decade, B cells have also been recognized as relevant participants within the immunological model of MS. Antibody-dependent (i.e., secreting intrathecal IgG) and -independent mechanisms are associated with B cell activity, both contributing to MS disease progression. Among antibody-dependent mechanisms, we mention the formation of autoantibodies that target specific CNS structures and the role of B cells in antigen presentation to T cells. Antibody-independent mechanisms consist of the secretion of cytokines and neurotoxic factors, and the formation of tertiary lymphoid organs and structures resembling germinal centers in the meninges of MS patients [110]. The role B cells play in BBB destruction in the context of MS has stimulated research in drug development; this has yielded potent disease-modifying drugs such as Rituximab [156], Ocrelizumab, and Ofatumumab [157], currently available on the market.

### 2.2. The BBB in Later Stages of MS—Focus on Neurodegeneration

**Figure 3:**
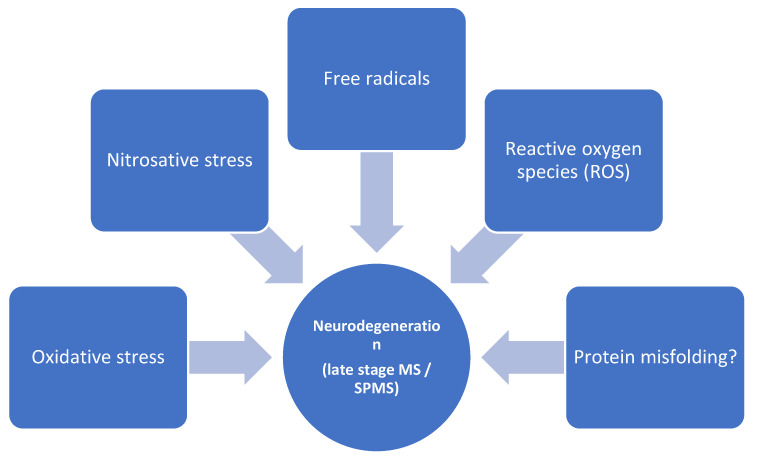
Potential mechanisms involved in neurodegenerative phases of multiple sclerosis.

Although encountered in smaller proportions in earlier stages of MS (including clinically isolated syndromes), neurodegeneration gains importance as the disease progresses, characterizing mainly the secondary-progressive form of MS (SPMS). The continuous advancement of disability in the absence of relapses is caused by slow but steady dendrite, axonal, and finally neuronal loss [158]. Though inflammation is considered to be the main factor responsible for cortical degeneration during the early stages of the disease [159], in the advanced stages, other complementary mechanisms come into action. Similar to other neurodegenerative diseases (AD, PD), the impact of oxidative stress, as well as the pathological production of free radicals and ROS, are relevant steps in sustaining neuronal loss (Figure 3).

The BBB remains a central structure that is altered as a result of neurodegenerative processes, subsequently leading to cortical damage. Oxidative stress alters the BBB structure, with brain ECs being the primary targets. According to a recent review [160], there are at least four different mechanisms that explain BMECs, known as the “oxidative stress multifaceted association”. The brain is a highly metabolic organ; glucose transport is higher across the BBB than across homologous structures located elsewhere in the human body, creating an environment that favors physiologically high amounts of ROS. Moreover, the presence of endothelial nitric oxide synthase generates high levels of nitric oxide, while the transport of fatty acids as an alternative energy source explains the generation of lipid peroxidation. Finally, the abundance of mitochondria at this level favors the production of reactive species, including superoxide, during the final stages of MS, which supports an older theory known as the “Mitochondrial Free Radical Theory of Aging” [161].

Free radicals, also known as pro-oxidants, are highly reactive, unstable chemical species that contain one or more unpaired electrons in their external orbitals [162]. Although now demonstrated to play a dual role (destructive and protective) in living systems, free radicals are mainly toxic byproducts of the aerobic metabolism which generate and sustain oxidative and nitrosative stress, finally leading to tissue damage [163]. Active inflammation in the CNS is associated with increased oxygen and nitrogen free radicals; however, the effects of these compounds in later stages of the disease, in the absence of acute demyelination, remain to be elucidated. Other molecular changes such as lipid peroxidation or protein alteration may also be a source of pro-oxidants, providing another link to the vicious cycle of neurodegeneration encountered in progressive MS [164].

Lastly, the potential role misfolded proteins play in MS pathology is a scarcely studied topic, albeit a relevant one. Misfolded protein aggregates are a common hallmark in other neurodegenerative disorders such as AD or PD, where amyloid plaques or hyperphosphorylated Tau protein have already become valuable biomarkers for diagnosis and therapeutic monitoring [165]. Moreover, the bidirectional relationship between Aβ and the BBB is of relevance from different perspectives, including Aβ clearance, BBB structural damage, and increased brain toxicity [166]. From this point of view, researchers have wondered if the pathological Aβ or Tau could also be involved in MS, mainly during later stages of the disease. In this context, one of the first studies conducted by Valis et al. [167], showed a significant increase in Aβ42 in the CSF of MS patients; however, no difference related to total and phosphorylated Tau protein levels was observed. Similarly, the work of David and Tayebi [168] demonstrated the presence of soluble amyloid oligomers in the brain tissue and cerebral spinal fluid of MS patients, although their exact role within this pathology is still to be determined. On the other hand, inconsistent results from several clinical trials [169,170] suggest that misfolded proteins alone are not the cause for BBB leakage during the neurodegenerative stages of MS, and that there are other—still unknown—pathological events occurring.

## 3. Conclusions

The role of NVU structures in MS onset and evolution has received increasing attention over the past few years. The amounting evidence strongly suggests these structures are involved in neuroinflammation and neurodegeneration—both hallmarks of MS.

Future studies should aim to uncover how various components of NVUs interact with their environment in the setting of disease. For example, finding an answer to the role astrocytes and microglia play in MS pathology should constitute a priority, and receive increased attention in MS research.

Achieving a comprehensive understanding of all structures, functions, and molecular underpinnings involved in MS will stimulate drug development to yield improved treatment options, which is ultimately the desired end result of research, awaited by clinicians and patients alike.

## Figures and Tables

**Table 1:** Physiological roles of the BBB and its dysfunctions in pathological conditions.

Physiological Roles of the BBB	BBB Dysfunctions in Pathological Conditions (including MS)
Maintaining of ionic metastasis	Ionic imbalance (neuronal hyperpolarization)
Facilitating brain nutrition	Impaired brain nutrition
Regulating levels of neurotransmitters	Neurotransmitter imbalance (pathologic inhibition/stimulation)
Limiting plasma macromolecules penetrating the brain	Macromolecule leakage
Protecting the brain against neurotoxins	Penetration of neurotoxins in the brain
Facilitating molecules elimination from the brain	Impaired residual products elimination

**Table 2:** Overview of alterations of the NVU components in the inflammatory state of multiple sclerosis.

NVU Components and Other Related Cells Encountered in MS Pathogenesis		Behavior in the Neuroinflammation Phase of MS	Most Relevant References
Brain microvascular endothelial cells		Reduction in the expression of TJ proteins Upregulation of different membrane receptors (i.e., Toll-like receptors) Increased production of adhesion molecules Increased pinocytosis Upregulation of glucose transporters (GLUT1) Downregulation of amino acid transporters (LAT1) Alterations of P-glycoprotein expression Degradation of the glycocalyx	[88,89,90,91,92,93,94,95,96]
Pericytes		Increased production of proinflammatory cytokines and chemokines Differentiation into fibroblasts or phagocytes Secretion of endothelial-disrupting factors	[97,98,99]
Astrocytes		Increased glutamate production Decreased production of protective factors (angiopoietin-1, Shh, IGF-1)	[100,101,102]
Oligodendrocytes/oligodendrocyte progenitor cells (OPCs)		Increased expression of inflammatory genes Increased phagocytic capacity Inhibition of OPC recruitment and maturation Increased apoptosis of oligodendrocytes Regional CNS demyelination	[53,103]
Microglia		Activation of microglia (M1 and/or M2 phenotype) Increased production of inflammatory mediators (IL-1, IL-6, TNF-α), chemokines (CCL2, CX3CL1, MIP-1), matrix metalloproteinases (MMPs), and oxygen free radicals	[104,105,106]
Neurons		Dendritic transection Axonal transection Apoptosis	[107]
Immune cells	Th1 CD4 cells	Secretion of IFN-γ and TNF-α	[108]
	Th17 CD4 cells	Production of IL-17, IL-21, IL-22	
	CD8 T cells	Direct cytotoxic effect Production of IFN-γ and IL-17	[109]
	B cells	Antibody-dependent mechanisms (secretion of intrathecal IgG) Antibody-independent mechanisms (secretion of cytokines, neurotoxic factors, formation of tertiary lymphoid organs)	[110]
	Neutrophils	Secrete pro-inflammatory cytokines (IL-1β, IL-6, IL-12, TNF-α, and IFN-γ)	[111]
	Monocytes	Change in phenotype (CD83 +, CD209 +) Promote the differentiation of Th1 and Th17 cells	[112]
	Mast-cells	Production of TNF-α	[113]

## Data Availability

All data and materials supporting the results of the present study are available in the published article.
